# Understanding Covalent Grafting of Nanotubes onto Polymer Nanocomposites: Molecular Dynamics Simulation Study

**DOI:** 10.3390/s21082621

**Published:** 2021-04-08

**Authors:** Seunghwa Yang

**Affiliations:** Mechanical Energy Engineering Division, School of Energy Systems Engineering, Chung-Ang University, Seoul 06974, Korea; fafala@cau.ac.kr; Tel.: +82-2-820-5266

**Keywords:** carbon nanotube, nanocomposites, molecular dynamics simulations, covalent grafting, solubility parameters

## Abstract

Here, we systematically interrogate the effects of grafting single-walled (SWNT) and multi-walled carbon nanotubes (MWNT) to polymer matrices by using molecular dynamics (MD) simulations. We specifically investigate key material properties that include interfacial load transfer, alteration of nanotube properties, and dispersion of nanotubes in the polymer matrix. Simulations are conducted on a periodic unit cell model of the nanocomposite with a straight carbon nanotube and an amorphous polyethylene terephthalate (PET) matrix. For each type of nanotube, either 0%, 1.55%, or 3.1% of the carbon atoms in the outermost nanotubes are covalently grafted onto the carbon atoms of the PET matrix. Stress-strain curves and the elastic moduli of nanotubes and nanocomposites are determined based on the density of covalent grafting. Covalent grafting promotes two rivalling effects with respect to altering nanotube properties, and improvements in interfacial load transfer in the nanocomposites are clearly observed. The enhanced interface enables external loads applied to the nanocomposites to be efficiently transferred to the grafted nanotubes. Covalent functionalization of the nanotube surface with PET molecules can alter the solubility of nanotubes and improve dispersibility. Finally, we discuss the current limitations and challenges in using molecular modelling strategies to accurately predict properties on the nanotube and polymers systems studied here.

## 1. Introduction

Over the past two decades, immense efforts have endeavored to leverage the unique properties of carbon nanotubes (CNTs) to design and manufacture multifunctional nanocomposites for vehicles, electronics, sensors, and other applications [1,2,3,4]. While CNTs are versatile, several challenges remain that have limited the CNT use for practical applications, some of which have attracted researchers in the field of nanostructured materials. Major issues include the agglomeration and bundling of dispersed CNTs (which arises because of differences in the solubility of the CNTs and host matrix) [5,6,7], intrinsically weak interfacial adhesion of CNTs to common polymers [8,9,10], waviness and kinks in the CNTs [11,12,13,14], and structural defects in CNTs (such as Thrower-Stone-Wales defects and vacancies) [15,16,17,18]. Efforts to overcome these challenges often aim to obtain a microstructure in which straight CNTs are well dispersed yet strongly bonded to a matrix, which would promote excellent load transfer between the CNTs and matrix.

The issue of CNT waviness and defects arise from the intrinsic properties of CNTs, while CNT dispersion in the polymer matrix and corresponding interfacial load transfer rely on interactions among the embedded CNTs and host. Solutions to the latter two factors are important to enable the practical applications and commercialization of CNT-based nanocomposites and are often approached chemically modifying the CNT surface [19]. Such chemical modifications can occur by bonding CNTs to surface functional groups on the polymer or other approaches, and surface modifications are classified into covalent and non-covalent grafting. Non-covalent grafting tailors interactions among CNTs and polymer matrices by exploiting conjugated polymers between them. On the other hand, covalent grafting involves needlework of CNTs, thus, can essentially break the conjugated structure of CNTs. Covalent grafting promotes stronger interfacial properties in CNT-polymer nanocomposites than non-covalent grafting and, thus, is the more widely used grafting method [20,21,22]. 

Nonetheless, it remains uncertain whether covalent grafting is an efficient method to improve the properties of CNTs-polymer nanocomposites. This is because covalent grafting promotes two competing effects: (1) improvements to interfacial load and phonon transfer and (2) alteration of CNT properties. Usually, it is difficult to experimentally measure the alteration of CNT properties post-grafting. Moreover, precise control over the surface functionalization of CNTs and pull-out displacement of CNTs should be implemented to assess the shear strength and other interfacial properties of the grafted composite. Most of the properties of nanocomposites are readily influenced by the extent of dispersion of the embedded CNTs, or other nanofillers. Thus, some of the improvements to the properties of CNT-grafted nanocomposites may be attributed to improved dispersion and intercalation of CNT into the polymer matrix. In this case, the two rivaling factors of improved interface and CNT alteration might balance each other. Thus, establishing a theoretical background to understand and evaluate the relative dominance of these three factors in preparing CNTs-polymer nanocomposites that have high performance, is still necessary.

Atomistic molecular dynamics (MD) simulations are effectively implemented to study a wide range of engineering polymer matrices and nanofillers. Thus, MD simulations are employed here to establish relationships between CNT-polymer structure and macroscopic properties. For instance, remarkable increases in the mechanical properties of nanosilica-polyimide composites have been demonstrated by using MD simulations [20]. The motion of the chains in the vicinity of the grafted nanoparticles enabled covalently grafted moieties to act as a rigid anchor that hinders the motion of molecules. Therefore, the covalently grafted moieties support the confined disentanglement of the matrix molecules and thus improved elastic moduli of the nanocomposites. The mechanical properties of silica nanoparticles, however, are not sensitive to the covalent functionalities because the silica nanoparticle has a filled solid circular configuration. MD simulations also demonstrated that covalent grafting could facilitate phonon transfer along the grafted interface, which can influence thermal transport across the interface between the CNT and polymer matrix [21]. Yet the thermal conductivity of CNTs is degraded by functionalization [22], which makes it important to consider both alteration of CNT properties and increased phonon transport when evaluating the effectiveness of covalent grafting. 

In retrospect, enormous efforts have been dedicated to understanding the mechanical properties of CNTs and to establish the structure-to-property relationship in nanocomposites. The multi-walled (MWNT) carbon nanotubes are more popular and cost-effective than the single-walled (SWNT) carbon nanotubes, thus, studies on strengthening and optimization of the inter-tubular interaction have been performed continuously. Since individual tubes are interacting with each other through the weak van der Waals (vdW) interactions, strengthening mechanism of the MWNT can be tailored by addressing some interfacial phenomena such as defects [23]. According to recent MD simulation studies [24,25], it has been proposed that controlling and optimization of the inter-tubular crosslinking can be a promising strategy to improve the strength of MWNT. Since external loads are transferred to the outermost CNT in MWNT from matrix, understanding the mechanical behavior of MWNT compared with the SWNT is essential to adopt the MWNT as multifunctional reinforcement.

The influence of covalent grafting on the interfacial properties of nanocomposites has been studied by predictive algorithms. However, computational studies into the dispersion of functionalized CNTs through the Hildebrand solubility parameter [26,27] have been relatively confined to designing solution processes and predicting the phase exfoliation of CNTs [6,26,27,28]. Even if the Hildebrand solubility parameter cannot be quantitatively correlated with physical properties of nanocomposites, it provides a useful measure of the extent of dispersion of CNTs in the polymer matrices. In order to use CNTs for smart composites sensors, dispersion of the CNTs is a critical issue to form a percolation network for efficient electron hoping for the sensors. Thus, the extent to which CNTs improve the multifunctionality of a matrix can be gleaned by comparing the solubility parameters of the CNTs and the matrix phase [6].

Here, we use MD simulations to systematically investigate the alterations in functionalized CNT properties, improvement of interfacial load transfer, and dispersibility of CNTs. We then use the insights from this study to evaluate the effectiveness and benefits of covalently grafting CNTs to the polymer matrix. Both SWNT and MWNT are considered, and polyethylene terephthalate (PET) is used as an amorphous polymer matrix. Various systems are modeled, including several with increasing extents of covalent grafting from the outermost side wall of the CNTs to the carbon atoms of the PET matrix via ethyl functional groups. The transversely isotropic elastic constants of the functionalized CNTs and nanocomposites are established from stress-strain curves. Finally, the Hildebrand solubility parameter of non-functionalized and CNT-functionalized PET matrices are compared and up-to-date techniques for modeling nanocomposites with periodic molecular unit cells are discussed.

## 2. Molecular Modeling and Simulation

### 2.1. Molecular Unit-Cell Model Construction

**Figure 1:**
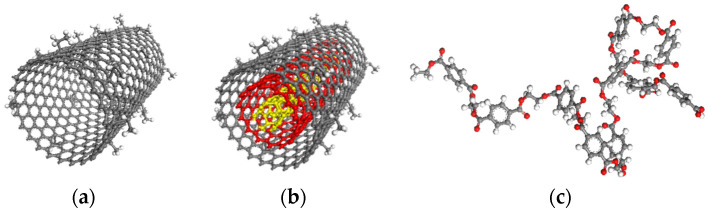
Molecular models of (**a**) (23, 0) single-walled carbon nanotubes (SWNT), (**b**) (23, 0), (14, 0), (5, 0) multi-walled carbon nanotubes (MWNT), and (**c**) polyethylene terephthalate (PET) chain.

Here, a SWNT with a chirality of (23, 0) and a MWNT consisting of three SWNTs with chiralities of (23, 0), (14, 0), and (5, 0) were modeled as a reinforcing phase. An amorphous PET was selected as the matrix phase. The commercially available molecular simulation program Material Studio was used for modeling molecular unit cells and geometry optimizations [29]. A polymer consistent force field (PCFF) [30] was used to describe all of the inter- and intra-atomic interactions. Each nanotube was surface-treated with ethyl functional groups that enable grafting to the PET matrix, as shown in Figure 1. Nanotubes were grafted by connecting either 0, 20, and 40 carbon atoms with the PET matrix each of which corresponds to 0%, 1.55%, and 3.1% of covalent grafting density, to enable detailed studies of grafting extent. Each nanotube was modeled with periodic boundary conditions along the longitudinal direction of the tube. Prior to embedding into the PET matrix, the nanotubes are optimized to their lowest potential energy state by using the conjugate gradient method. 

**Figure 2:**
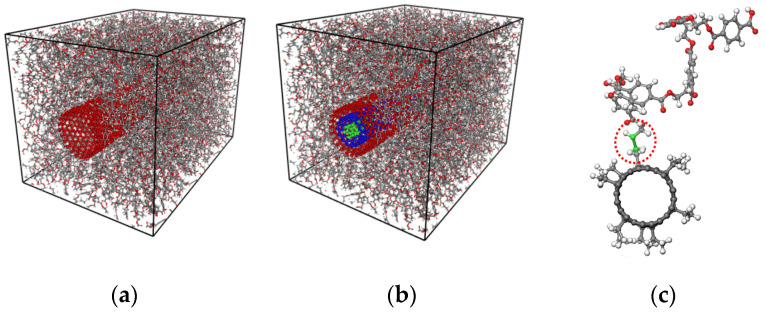
Molecular models of (**a**) grafted SWNT/PET nanocomposites, (**b**) grafted MWNT/PET nanocomposites, and (**c**) covalent grafting between the carbon nanotubes (CNT) and PET chain.

The PET matrix for the construction of nanocomposites is consists of 16 chains, each of which is polymerized by 30 ethylene terephthalate monomers. Periodic boundary conditions are also applied to the PET unit cell. The total potential energy of the PET unit cell structure is minimized by the conjugate gradient method. SWNTs and MWNTs with different numbers of surface ethyl functional groups were embedded into the PET matrix to construct the transversely isotropic nanocomposites unit cell structures, as shown in Figure 2a,b. The periodic boundary condition is applied along the longitudinal direction of the embedded nanotubes, thus, equivalent continuum model of the nanotube is infinite cylinder. For convenience, the longitudinal direction of the embedded nanotube was set as the *x*-axis in a Cartesian coordinate system for the nanocomposites and nanotubes. Prior to covalently grafting the nanotubes to the matrix, nanocomposites unit cells were again optimized by the same minimization scheme. The compositions of the unit cell for all of the molecular structures are summarized in Table 1. The pure PET unit cell is considered as a reference structure. 

The covalent grafting process is based on a dynamic cross-linking process or virtual in-situ cross-linking process that is widely used in molecular modeling of thermoset epoxy [31,32,33]. First, candidate atoms in the ethyl functional groups on the surface of the nanotubes and in the PET chains are defined. In the nanotubes, all of the terminal carbon atoms in the ethyl groups were selected as candidate atoms for the grafting process. In the PET matrix, all of the carbon atoms, except aromatic carbon atoms in benzene rings, were chosen for grafting. To develop covalent grafting, a cut-off distance is defined before close-contact monitoring. Here, the initial cut-off distance was set at 3 Å. After energy minimization, all of the close-contact distances between the candidate atoms in the carbon nanotube and PET matrix are monitored and compared with the cut-off distance. If the distance between candidate atoms falls within the cut-off distance, a new covalent bond is formed along with the corresponding valence and atomic charge modifications according to the structure shown in Figure 2c. Unless all the candidate atoms are grafted, the cut-off distanced increases by 1 Å and the combination of the close contact monitoring, grafting, and valance modification processes are repeated until the target grafting density is reached. Notably, the dynamic cross-linking process is not reactive but generates stable cross-linked structures.

### 2.2. Equilibration and Production Run for Nanocomposites

Both an equilibration and production run were performed by using the large-scale atomic/molecular massively parallel simulator (LAMMPS) program [34]. The optimized nanocomposites and PET structures were equilibrated at 300 K and 1 atm through an isothermal-isobaric (NPT) ensemble simulation prior to conducting production runs. The temperature and pressure were controlled by using a Nosé–Hoover extended Hamiltonian method [35,36] with a damping constant of 20 femtoseconds (fs) for 3 nanoseconds (ns). All of the equations of motions are solved every 1 fs by using the Verlet algorithm for time integration. During the equilibration process, the pressure tensor components are anisotropically controlled. As a result, the final volume fraction of the nanocomposites after 3 ns of equilibration is slightly different from the initial volume fraction assigned in the model.

After equilibration, the stress-strain curves of the nanocomposites were determined in a production run. To predict the tensile stress-strain curves, each unit cell was quasi-statically elongated at a constant true strain rate of 0.0002 ps^−1^ until the true strain of the structures reached 6%. In tensile deformation, atoms at the boundary of the unit cell are displaced together with the elongation of the unit cell. Therefore, the increased interatomic potential energy in the displaced atoms act as driving force to increase the internal stress of the nanocomposites. The internal pressure tensor components perpendicular to the tensile loading direction were kept at 1 atm to account for the Poisson’s effect, and the virial stress components are recorded at every 2000 steps to predict the stress-strain curves. The tensile tests were repeated three times by assigning a different random number seed for establishing the initial velocity distribution of the atoms in a unit cell. Thus, three and six stress-strain curves were obtained for the longitudinal tension and transverse tension of nanocomposites, respectively. The elongation along *x*-axis results in longitudinal tension and those along the *y* and *z* axes yield transverse tension. The stress-strain curves were averaged in each loading case for comparison.

The shear stress-shear strain relations were determined in a similar manner. Unit cells are quasi-statically distorted at the same strain rate. NVT ensemble simulations were used to determine and monitor the shear stress components from the virial theorem at each shear strain step to complete the shear stress-shear relations. As the nanocomposite unit cell is transversely isotropic, the shearing test on the *xy* and *xz* planes yield longitudinal shearing and that on the *yz* plane results in in-plane shearing. The shear tests were also repeated three times on each plane. The longitudinal shear stress-strain relation was averaged over six stress-strain curves and the in-plane shear stress-strain relation was averaged over three stress-strain curves. All of the tensile and shearing tests were implemented at 300 K.

### 2.3. Molecular Mechanics Calculation to Establish Nanotube Properties

The elastic constants of SWNT and MWNT grafted at their side walls at varying numbers of sites were determined to understand the altered properties of functionalized nanotubes. As both SWNT and MWNT nanotubes have tubular structures and different spatial configurations of carbon atoms (specifically in armchair and zigzag organization), we assume that the nanotubes are transversely isotropic [10,37,38]. Both nanotubes are much stiffer than the PET matrix and their elastic stiffness is insensitive to temperature [39]. Thus, molecular mechanics (MM) calculations were used to establish the elastic constants of SWNT and MWNT. Moreover, we modeled both nanotubes as solid cylinders to define the strain energy density. 

**Figure 3:**
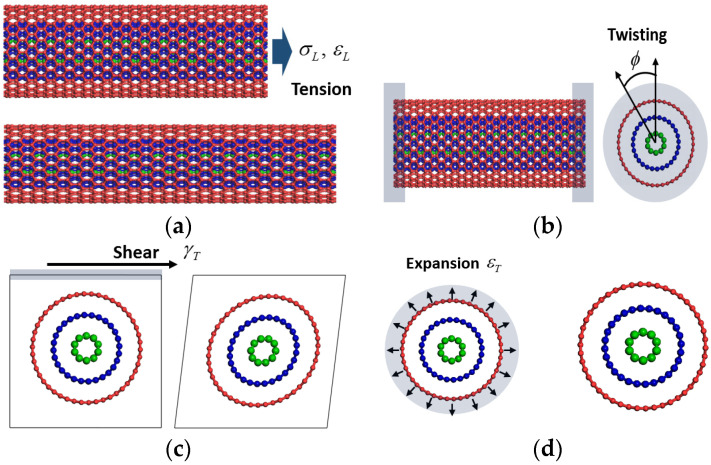
Molecular mechanics calculation scheme for transversely isotropic elastic constants of SWNTs and MWNTs. Schematics that illustrate the (**a**) longitudinal modulus and major Poisson’s ratio, (**b**) longitudinal shear modulus, (**c**) in-plane shear modulus, and (**d**) in-plane bulk modulus.

The elastic constants of the nanotubes were determined based on the relationship between the elastic constant and strain energy density in the MM calculations. The longitudinal Young’s modulus and major Poisson’s ratio of both nanotubes were calculated from a quasi-static longitudinal tension test of the nanotube unit cell model, as shown in Figure 3a. At each loading step, the corresponding elastic strain energy density was calculated after geometry optimization process that employed the conjugate gradient method. The longitudinal Young’s modulus *E_L_* was established by the following relationship:

(1) $$u_{E_{L}}=\frac{E_{L}\varepsilon_{L}^{2}}{2}$$

where $u_{E_{L}}$ is the strain energy density in longitudinal tension, and $\varepsilon_{L}$ is the elastic strain along the longitudinal direction of the nanotubes. The major Poisson’s ratio was calculated by using the longitudinal and transverse strain via the following equation:

(2) $$\nu_{LT}=-\frac{\varepsilon_{T}^{}}{\varepsilon_{L}^{}}$$

where $\varepsilon_{T}^{}$ is the transverse strain component in longitudinal tension.

Nanotubes were twisted as shown in Figure 3b to establish the longitudinal shear modulus. In contrast to the longitudinal tension where periodic boundary conditions were applied in the longitudinal direction of the nanotubes, finite nanotubes are modeled for the twisting and longitudinal shear modulus. Atoms in one edge of nanotubes are fully constrained to impose a twisting angle to the nanotubes stepwise. At each step of twisting, atoms in the other edge of nanotubes are quasi-statically displaced based on the defined twisting angle and the total energy of unconstrained atoms in nanotubes was subsequently optimized. The longitudinal shear modulus $G_{L}^{}$ of the nanotubes was determined by the relationship between the twisting angle and torsional deformation energy, as given by:

(3) $$U_{G_{L}}=\frac{G_{L}^{}\phi^{2}J}{2L}$$

where ϕ is the twisting angle, *J* is the polar moment of inertia, and *L* is the length of the nanotube. For the MWNT, all of the inner and outer tubes were simultaneously twisted.

Both the in-plane shear modulus and in-plane bulk modulus were calculated from the in-plane projection of the carbon atoms in a periodic unit cell according to the defined in-plane strains. At each strain increment, the nanotubes were displaced, and the corresponding deformation energy density was monitored. As distinct from the longitudinal tension and twisting, geometric optimization processes were not implemented to retain the deformed configurations. The in-plane shear modulus *G_T_* was obtained from the following linear elastic relationship:

(4) $$u_{G_{T}}=\frac{G_{T}\gamma_{T}^{2}}{2}$$

where $u_{G_{T}}$ is the distortional energy density and $\gamma_{T}^{}$ is the applied in-plane shear strain of the nanotube. The in-plane bulk modulus was obtained by applying the following equation:

(5) $$u_{K_{T}}=2K_{T}\varepsilon_{T}^{2}$$

where is $u_{K_{T}}$ is the in-plane dilatational energy density and $\varepsilon_{T}$ is the normal strain applied radially to the nanotubes. The elastic strain applied to the nanotubes was within 5% in all of the MM calculations. The volume of the nanotubes was determined from the connoly surface of the carbon atoms that constituted each nanotube at each loading step.

## 3. Results and Discussions

### 3.1. Elastic Moduli of SWNTs and MWNTs

The transversely isotropic elastic constants of SWNTs and MWNTs are listed in Table 2 and Table 3, respectively. For both nanotubes, all of the elastic constants decrease as the number of grafting sites in outermost sidewall of the tube increases. This arises from the *sp*^3^ hybridization of carbon atoms in CNTs that are bonded by ethyl functional groups. As the carbon atoms functionalized with ethyl groups for the covalent grafting are *sp*^3^ hybridized, partially double-bonded primary interaction between the functionalized carbon atoms and their neighboring atoms are degraded to the single bonds. Therefore, covalently grafted CNTs become less stiff than pristine CNTs. The alteration of nanotube properties as a result of covalent grafting presents a trade-off for improved interfacial load transfer from the PET matrix to nanotubes and vice versa. As the macroscopic properties of nanocomposites are readily influenced not only by the properties of nanotubes but also the interfacial load transfer mechanism, the results in Table 2 and Table 3 suggest the potential for an optimal number of grafting sites or grafting ratio. 

Analysis of Table 2 and Table 3 also reveals that MWNTs have superior elastic constants when compared to SWNTs. As the displacement boundary conditions are applied to each concentric tube, the MWNTs have larger deformation energy densities than SWNTs at the same external strain. Moreover, the elastic constants of MWNT are less sensitive to the grafting density than those of SWNTs. As the outermost tube in MWNTs is covalently grafted to the PET matrix, the number of *sp*^3^ hybridized carbon atoms in each MWNT is lower than in SWNTs. Thus, the deformation energy density, or deformation energy and resultant elastic constants, of MWNTs are less sensitive to the number of grafting sites than those of SWNTs. Meanwhile, the SWNT is hollow and has a lower mass than MWNTs, which can be advantageous. 

The transversely isotropic fourth-order elastic stiffness tensor can be constructed by applying the constants in Table 2 and Table 3 to the linear elasticity theory of transversely isotropic materials, as defined by the following equations:

(6) $$\begin{array}{l} E_{L}=C_{11}-\frac{2C_{12}^{2}}{C_{22}+C_{23}^{}}\\ E_{T}=C_{22}-\frac{C_{12}^{2}\left(C_{22}-2C_{23}\right)+C_{11}C_{23}^{2}}{C_{11}C_{22}-C_{12}^{2}}\\ G_{T}=\frac{1}{2}\left(C_{22}-C_{23}\right)\\ K_{T}=\frac{1}{2}\left(C_{22}+C_{23}\right)\\ \nu_{LT}=\frac{C_{12}^{}}{C_{22}+C_{23}^{}}\\ G_{L}=C_{55}^{}=C_{66}^{} \end{array}$$

where *C_ij_* is the engineering notation for the elastic stiffness component. The full elastic stiffness tensors of the SWNT and MWNT enable the prediction of the elastic stiffness of nanocomposites predicted from existing micromechanical models, such as the Mori-Tanaka (M-T) model, self-consistent (SC) model, double-inclusion (D-I) model, and others. In Section 3.3 of this manuscript, the Mori-Tanaka model is applied to provide a reference elastic stiffness of the nanocomposites under a perfect interface condition using the the stiffness of grafted nanotubes and pure PET matrix. The elastic stiffness matrix components of SWNT and MWNT are arranged in Table 4.

### 3.2. Stress-Strain Relationships in Nanocomposites

**Figure 4:**
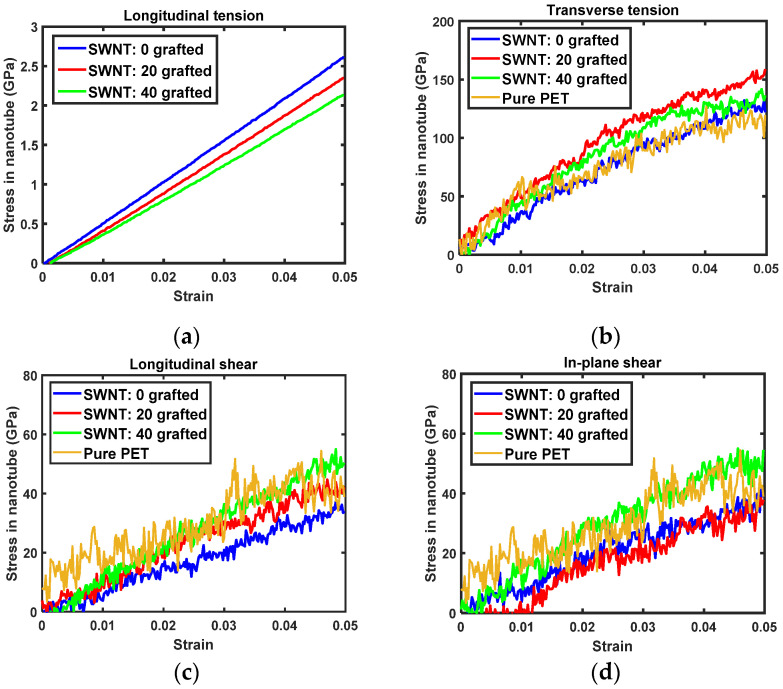
Transversely isotropic stress-strain relation of SWNT/PET nanocomposites: (**a**) longitudinal tension, (**b**) transverse tension, (**c**) longitudinal shearing, (**d**) in-plane shearing.

**Figure 5:**
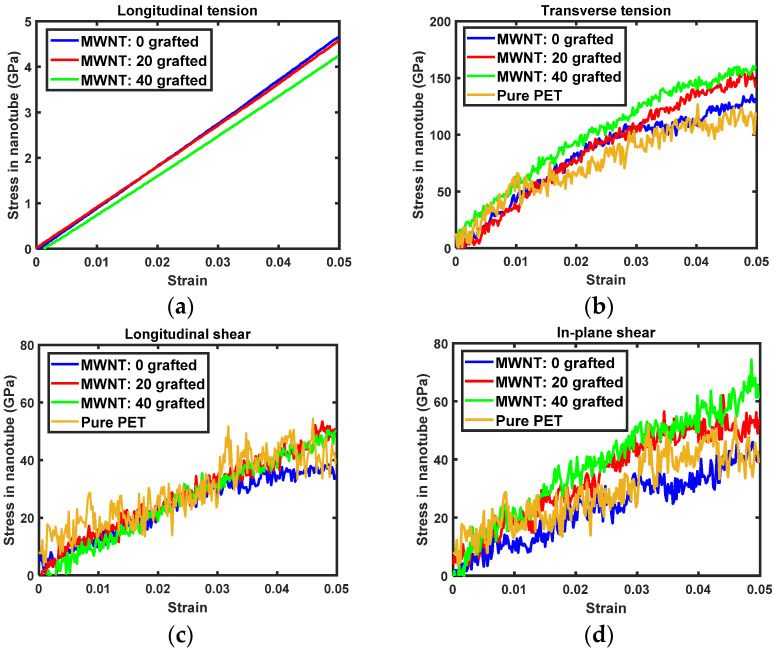
Transversely isotropic stress-strain relation of MWNT/PET nanocomposites: (**a**) longitudinal tension, (**b**) transverse tension, (**c**) longitudinal shearing, (**d**) in-plane shearing.

The transversely isotropic stress-strain curves of PET nanocomposites reinforced with either SWNTs and MWNTs are shown in Figure 4 and Figure 5. For longitudinal tension, the stress in the nanocomposite decreases as the number of covalent grafting sites in nanotubes increases. Reduced longitudinal tension is mainly attributed to the alteration of both nanotubes by the *sp*^3^ hybridization of grafted carbon atoms on the nanotubes. The periodic boundary condition is applied in the longitudinal direction of the nanotubes to determine the longitudinal tension of the nanocomposites. As such, the alteration of the nanotube directly relates to the behavior of nanocomposites. As the molecular model used here includes perfectly aligned nanocomposites, the stress-strain curves shown in Figure 4 and Figure 5 provide the upper bound of the nanocomposites Young’s modulus at each covalent grafting.

In transverse tension, the transverse stress of the nanocomposites increases with the number of covalent grafting sites, as shown in Figure 4b and Figure 5b. For composites with pristine nanotubes (i.e., without grafting), the transverse stress-strain relationship shows that nanocomposites do not exhibit any reinforcing effects versus pure PET. This result demonstrates that interfacial load transfer between the pristine nanotube side wall and surrounding PET matrix molecules is insufficient to influence mechanical properties [10,40]. However, nanocomposites with grafted nanotubes show increased levels of stress tolerance. At 5% of transverse stress, nanocomposites with 40 grafted nanotubes show nearly 50 MPa of improvement in load-bearing capacity versus pure PET. As the transverse elastic modulus was reduced by covalent grafting, the elevated transverse stress of the nanocomposites is attributed to improved interfacial load transfer via the covalent bond between the nanotube and PET matrix.

The same tendencies are observed for longitudinal and in-plane shearing. Namely, the shear stress in the composites increases with increased numbers of grafting sites. There was initial residual stress at the beginning of in-plane shearing of MWNT/PET composites. Thus, the in-plane shear stress of the nanocomposites was calibrated based on the initial shear stress to enable more accurate comparisons of the evolution of shear stress with in-plane shear strain. As both longitudinal and in-plane shear moduli decrease with nanotube grafting, improvements in shear stress-shear strain relationship (Figure 4c,d and Figure 5c,d) reflect greater interfacial shear load transfer capacity arising from the covalent bond between the nanotubes and PET matrix. 

### 3.3. Elastic Constants of Nanocomposites

Quantitative insights into the effects of covalent grafting on the transversely isotropic Young’s moduli and shear moduli of the nanocomposites were established from the stress-strain curves, as shown in Figure 4 and Figure 5, within 3% of the elastic strain. The isotropic Young’s modulus and shear modulus of pristine PET were also determined within 3% of strain and were 2.61 GPa and 0.90 GPa, respectively. As covalent grafting tailors the interface between the nanotubes and PET matrix, the reference elastic moduli of the nanocomposites must be predicted by using models that include perfect interfacial conditions. Thus, the M-T model was used to predict the transversely isotropic elastic moduli of nanocomposites at the same volume fraction of the nanocomposites used in the molecular models. The fourth-order elastic stiffness tensor C of the nanocomposites is provided in linear algebraic form [41,42] as:

(7) $$C=\left(f_{m}C_{m}+f_{p}C_{p}:A_{p}\right):{\left(f_{m}I+f_{p}A_{p}\right)}^{-1}$$

where C is the fourth-order stiffness tensor, *f* is the volume fraction of the constituent, and the subscripts *p* and *m* indicated the filler (the CNT) and matrix (the PET), respectively. The fourth-order tensor $A_{p}$ is the dilute strain concentration tensor of the filler, and is defined as:

(8) $$A_{p}={\left[I+S:C_{m}^{-1}:\left(C_{p}-C_{m}\right)\right]}^{-1}$$

where I is the identity tensor and S is the Eshelby tensor of ellipsoidal inclusion that reflects perfectly bonding to the host material [43]. The closed form solution of the Eshelby tensor can be found in reference [42]. In the M-T model, the equivalent inclusion of the CNTs is aligned identically shaped infinite cylinder with transversely isotropic elastic symmetry, thus, the effective elastic constants of the nanocomposites determined from Equations (7) and (8) lie on or within the Hashin-Shtrikman bounds [42]. 

**Figure 6:**
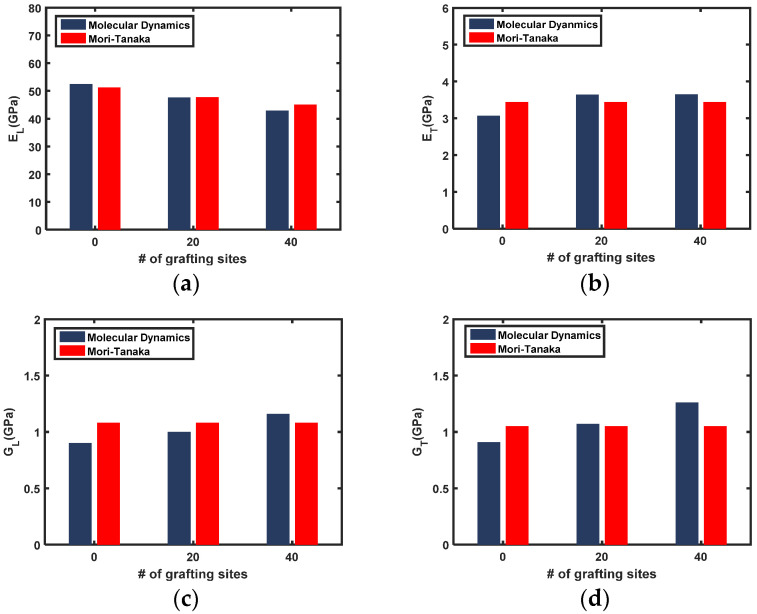
Transversely isotropic elastic modulus of SWNT/PET nanocomposites established by MD simulations or the M-T model. Calculated parameters of (**a**) longitudinal Young’s modulus, (**b**) transverse Young’s modulus, (**c**) longitudinal shear modulus, and (**d**) in-plane shear modulus obtained by both methods are plotted for nanocomposites with various numbers of grafting sites.

**Figure 7:**
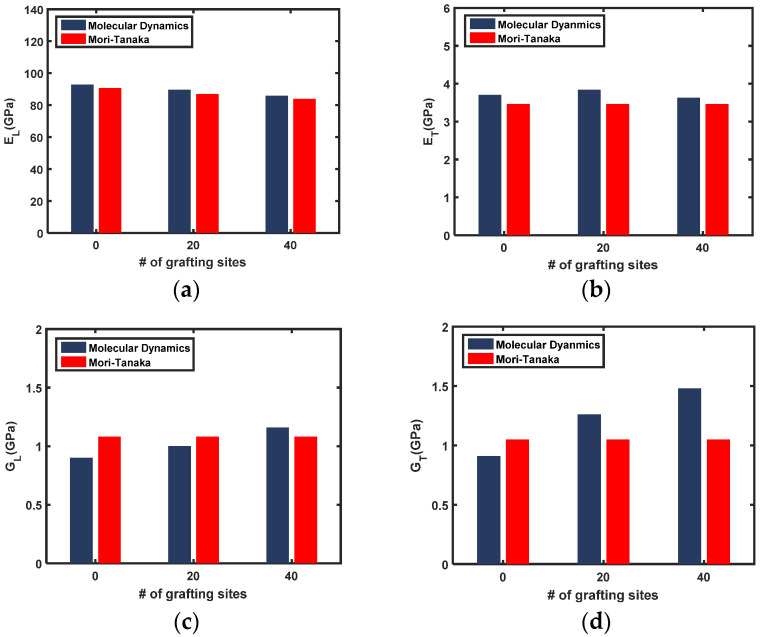
Transversely isotropic elastic modulus of MWNT/PET nanocomposites determined from the MD simulations and the M-T model: (**a**) longitudinal Young’s modulus, (**b**) transverse Young’s modulus, (**c**) longitudinal shear modulus, (**d**) in-plane shear modulus.

Comparisons of both the transversely isotropic Young’s moduli and shear moduli established by MD simulations and Equation (7) are provided in Figure 6 and Figure 7 for the SWNT and MWNT nanocomposites, respectively. The longitudinal Young’s modulus of both nanocomposites decreases as the number of grafting sites increases. The MD simulation results are generally slightly higher than those of the M-T model, which is attributed to the formation of a highly densified interphase zone that surrounds the nanotubes [10,44]. While the contribution of the interphase zone is accounted for in the MD simulation, the two-phase M-T model prediction does not consider the formation of an interphase zone. In this case, the multiphase homogenization method [45,46] or two-step homogenization approaches [47] are appropriate to account for the interphase zone.

The transverse Young’s modulus and shear moduli of the nanocomposites increase with the number of grafted sites. Notably, the MD simulation results are lower than those predicted by the M-T model for nanocomposites with non-grafted nanotubes. Even if the interphase zone forms around the nanotube, the load transfer between the interphase zone and pristine nanotubes via secondary bonding is insufficient to influence composite properties. Thus, the contribution of the interphase zone to the overall elastic moduli is not apparent. As the number of grafting sites increases, however, the trend reverses. The MD simulation results are greater than those predicted by the M-T model. The stiffness of the interphase zone becomes important because the interfacial load transfer between the nanotube and interphase zone is promoted by covalent bonds. This result also demonstrates that covalently or non-covalently grafted nanotubes, as well as nanotube surface functionalization, are important for exploiting the interphase zone to reinforce the nanocomposite. Alteration of the nanotube properties is inevitable for the covalent grafting, but the improved load transfer and participation of the interphase zone in load-bearing is anticipated to be beneficial.

In order to use the CNTs for hybrid composites sensors, interfacial load transfer from matrix to CNT or vice versa is an important parameter to increase the mechanical sensing ability. In randomly dispersed CNTs percolation networks for the sensors, interfacial load transfer is mostly by the shearing between CNT and surrounding matrix. Even if polydispersed CNT microstructure was not considered in our model, we can reasonably conjecture that the covalent grafting can improve the sensing capability of CNTs-based smart composites sensors. 

### 3.4. Concentration of Stress in Nanotubes

**Figure 8:**
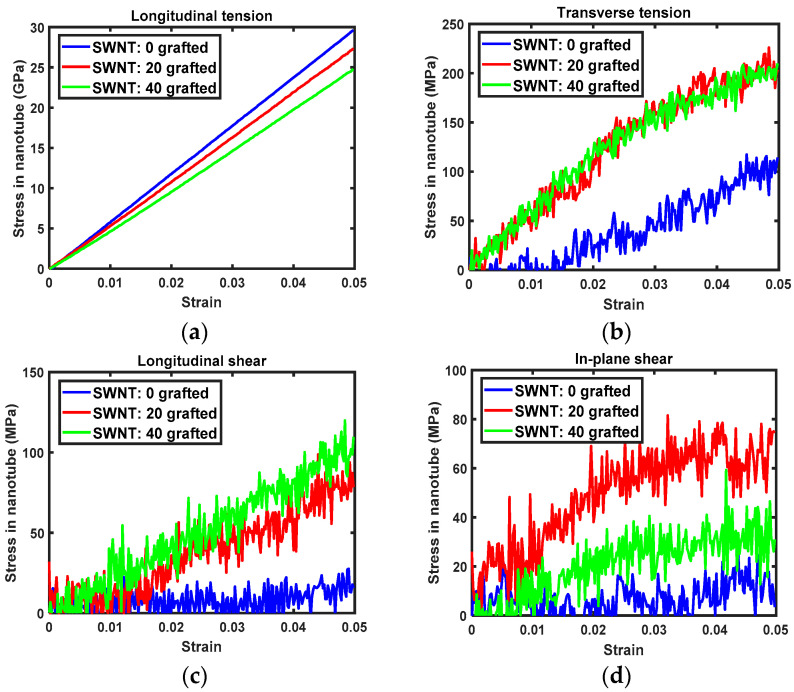
The evolution of stress of SWNTs in nanocomposites, specifically the (**a**) longitudinal tension, (**b**) transverse tension, (**c**) longitudinal shearing, and (**d**) in-plane shearing.

**Figure 9:**
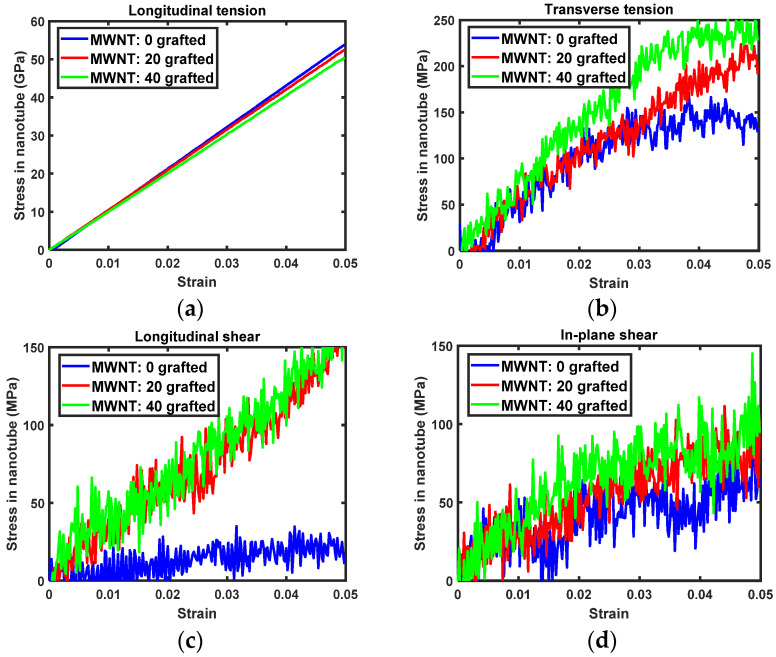
Stress evolution of MWNTs in nanocomposites, specifically the (**a**) longitudinal tension, (**b**) transverse tension, (**c**) longitudinal shearing, and (**d**) in-plane shearing.

Local stresses to the nanotubes were calculated according to the strain applied to the nanocomposites, to correlate improvements in the transverse Young’s modulus and shear moduli of nanocomposites with the interfacial load transfer in the PET-nanotube composites. Figure 8 and Figure 9 show the stress in SWNTs and MWNTs upon tension and shearing of nanocomposites. For longitudinal tension, the applied axial load is directly transferred to the nanotubes. In this case, the axial stress in both nanotube types decreases as the number of covalent grafting sites increases. The slopes of the axial stress-composite axial strain relation for the CNTs shown in Figure 8a and Figure 9a are almost identical to the longitudinal Young’s moduli of SWNTs and MWNTs.

For transverse tension, the stress in both nanotubes is on the order of ~10^2^ MPa, as shown in Figure 8b and Figure 9b. Notably, the transverse stress in grafted nanotubes increases much more rapidly than in pristine nanotubes despite the grafted tubes having lower elastic moduli. This inconsistency, which is shown in Figure 8 and Figure 9, is attributed to interfacial load transfer. By covalently grafting the nanotubes to the PET matrix, the applied transverse strain to the matrix phase in the composites eventually transfers to the nanotube via the interface. Thus, the improved interfacial load and displacement transfer via covalent grafting dominates the altered properties of nanotubes with respect to *sp*^3^ hybridization.

For longitudinal and in-plane shearing, the contribution of covalent grafting is clearly demonstrated. Pristine nanotubes carry almost no shear stress, with the exception of in-plane shearing for the MWNT/PET, as shown in Figure 8c,d and Figure 9c,d. As the covalent grafting increases, the shear stress concentrated to the nanotube increases prominently. This result supports the fact that the intrinsic adhesion between pristine nanotube and typical engineering polymer is weak in nature and can be dramatically improved by addressing the covalent grafting. Therefore, the improved shear moduli observed from the grafted nanocomposites was more dominantly affected by the improved interfacial shear load transfer than the degraded shear moduli of nanotube.

Comparing Figure 8d and Figure 9d, it is interesting that the shear stress concentrated to the MWNTs is larger than that concentrated to the SWNTs, even for the pristine MWNT. The superior shear stress transfer between MWNT and PET matrix to the SWNT is attributed to the improved vdW interaction at the interface between each tube and PET matrix as well as strong inter-tubular vdW interaction between inner and outer tubes. For the MWNT, three nanotubes interact with the PET matrix at the interface via vdW interactions. Therefore, the interface with the PET matrix is stronger than that in the SWNT-PET nanocomposites. Meanwhile, the two inner tubes in MWNT are pristine. As is shown in Figure 8d, interfacial shear load transfer between pristine nanotube and PET matrix is not prominent. Moreover, stress concentration to the grafted MWNT is more prominent than to the SWNT. Therefore, it can be concluded that the vdW interaction between each tube enables efficient shear load transfer from matrix to the inner nanotubes. On static and dynamics analysis of freestanding MWNT through equivalent shell theory, the vdW interaction between each tube plays very important role. [48,49,50]. According to ref. [48,49], the vdW interaction is dependent on the radius of the tube, especially when the radius is less than 7 nm. In our MWNT, diameter of the largest tube (23,0) is 1.8nm. Therefore, the vdW interaction between each nanotubes strongly affects the elasticity of MWNT in shearing of nanocomposites. In summary, the evolution of the stress in nanotubes shows that MWNTs are superior to SWNTs with respect to interfacial load transfer.

### 3.5. Nanotube Solubility and Dispersibility

The dispersion of nanotubes in the polymer matrix is challenging to control but nonetheless important for reinforcing the composite. The contributions of covalent grafting to the dispersibility of the grafted nanotubes are studied by using the Hildebrand solubility parameter [26] of the nanotubes and PET matrix. Materials with more similar solubility parameters are more miscible. The solubility parameter is defined by the following equation:

(9) $$\delta =\sqrt{c}=\sqrt{\frac{\Delta H-RT}{V_{m}}}$$

where *c* is the cohesive energy density, ΔH is the heat of vaporization, *R* is the gas constant, *T* is the temperature of the system, and *V_m_* is the molar volume of the system. Two parametric sets were prepared to understand the effects of covalently grafted PET functional groups on the solubility parameter. Specifically, one set varied the number of surface-functionalized ethylene terephthalate monomers on the outer most wall of the MWNT, and the other set varied the number of monomer groups on the grafted PET moieties. 

**Figure 10:**
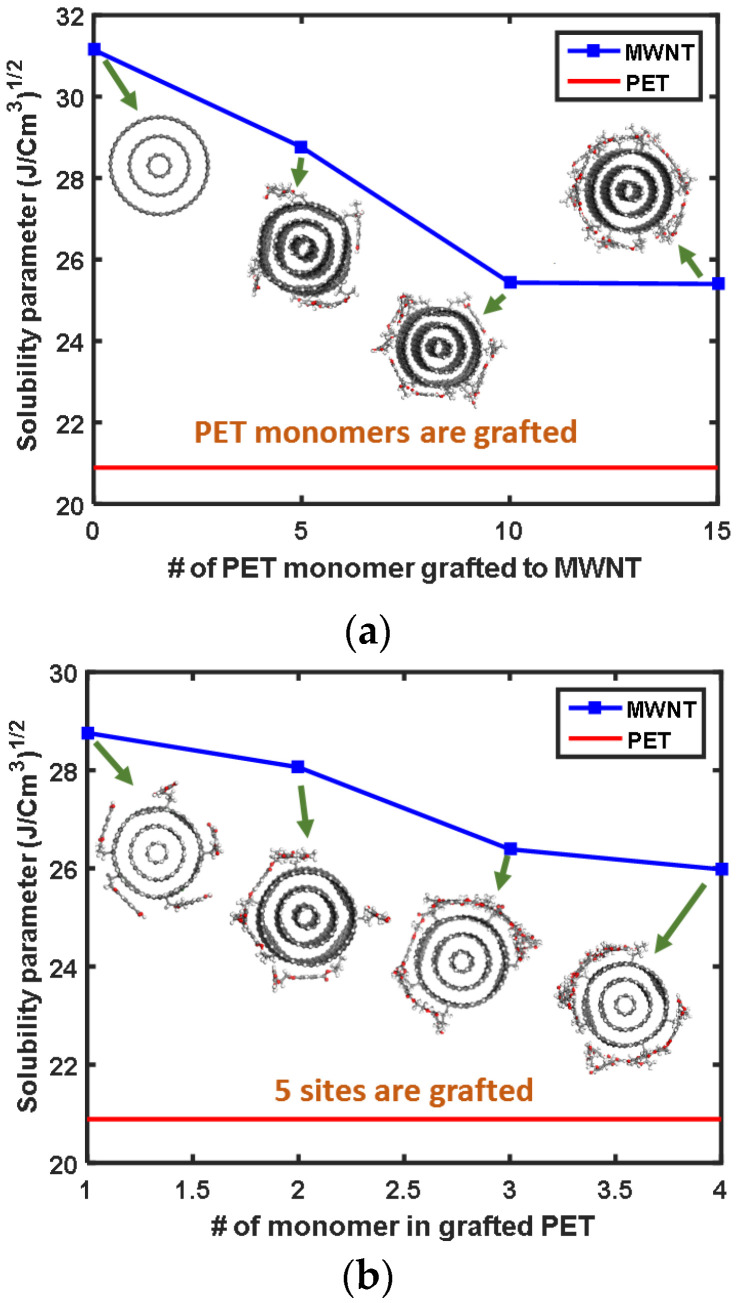
Hildebrand solubility parameters for MWNT and PET obtained by varying the (**a**) number of surface-functionalized ethylene terephthalte monomer and (**b**) number of PET monomer groups on the grafted PET moieties.

The solubility parameter of PET-functionalized MWNTs is compared with that of pure PET in Figure 10. The solubility parameter of PET-functionalized MWNT gradually decreases, nearing the solubility parameter of PET, as the number of covalently functionalized ethylene terephthalate increases (Figure 10a). The solubility parameter of pristine MWNTs is nearly 10$\sqrt{J/Cm^{2}}$ different than that of pure PET. Thus, MWNTs are not well dispersed in the PET matrix. By comparison, functionalizing MWNTs with PET monomers improves the miscibility of MWNT and PET. The solubility parameter of PET-functionalized MWNT becomes closer to that of PET when longer PET molecules are grafted (Figure 10b). Thus, the covalent grafting of PET to the surface of MWNT can improve the dispersion of nanotubes in the PET matrix and the intercalation of PET molecules into the gallery between exfoliated nanotubes.

The improved dispersibility of MWNTs by covalent grafting prompts a reconsideration of the molecular unit cell model in Figure 2. In the molecular model used in this study, and other MD simulation studies [10,38,51,52], it is assumed that the pristine nanotube is well dispersed and aligned in the polymer matrix. Accounting for randomly oriented and aggregated CNTs requires that several short nanotubes need to be embedded in the polymer matrix in the simulation model and, additionally, a long equilibration time. The stable surface structures of pristine CNTs make them difficult to disperse into PET matrix, as evidenced by the solubility parameter. In this respect, the well-dispersed unit cell model shown in Figure 2a is an idealized model for CNT-embedded nanocomposites. Even if the transverse elastic moduli of well-dispersed nanocomposites unit cell models are directly compared, the effect of locally aggregated CNTs should accounted for by more rigorous evaluation of the contributions of covalent grafting. The study of aggregated nanotubes in polymer matrices involves mesoscale simulations that incorporate coarse-graining, and we are considering this simulation route as future work.

## 4. Conclusions

Here an MD simulation was performed to evaluate the effects of covalent grafting of nanotubes on the elastic properties of PET-nanotube nanocomposites. When SWNTs and MWNTs were covalently grafted to a PET matrix, *sp*^2^ hybridization occurs and the transversely isotropic elastic constants of both nanotubes decrease. This reduction demonstrates that covalent grafting of nanotubes can play a negative role.

In terms of elastic moduli, covalent grafting of nanotubes dramatically improved interfacial load transfer of nanocomposites for transverse tension and shearing. The transversely isotropic elastic moduli of nanocomposites were determined and compared with the predictions by the M-T model, which assumes perfect interfacial bonding conditions. As the extent of covalent grafting increases, the MD simulation results of transverse tension and shearing substantially increase and exceed the M-T model solution, due to the contributions of a highly densified interphase zone.

Examination of the evolution of internal stress in nanotubes for tension and shearing of the nanocomposites revealed covalent grafting as a dominant factor for improved interfacial load transfer. Due to the slender configuration of the nanotube, improvements in load transfer were greater under shearing conditions. Moreover, the MWNT showed stronger intrinsic interfacial adhesion strength than the SWNT due to its robust characteristics with respect to transverse tension and in-plane shearing. 

Interrogations with the Hildebrand solubility parameter determined that covalent grafting enhanced the dispersibility of nanotubes in a PET matrix. Functionalizing longer and more PET to the surface of nanotubes results in greater miscibility to the PET matrix. As facile interpenetration of PET molecules into the CNTs results in the sufficient formation of a polymer sheathed zone, covalent grafting contributes to interfacial properties as well as the formation of an interphase zone.

## Figures and Tables

**Table 1:** Unit-cell composition of nanocomposites.

	Carbon Nanotubes		Nanocomposites		
System	Length (Å)	Volume Fraction	Y = Z (Å)	X (Å)	Density (g/cm^3^)
SWNT: 0	30.59	6%	64.90	30.59	1.29
SWNT: 20					1.30
SWNT: 40					1.32
MWNT: 0	30.94		65.63	30.94	1.33
MWNT: 20					1.37
MWNT: 40					1.40

**Table 2:** Transversely isotropic elastic constants of SWNTs [GPa].

SWNT	*E_L_*	*K_T_*	*G_L_*	*G_T_*	*v_LT_*
Pristine	603	260	300	105	0.43
20 grafted	560	197	277	99	0.39
40 grafted	527	186	241	96	0.33

**Table 3:** Transversely isotropic elastic constants of MWNTs [GPa].

MWNT	*E_L_*	*K_T_*	*G_L_*	*G_T_*	*v_LT_*
Pristine	1089	384	371	190	0.44
20 grafted	1044	372	350	184	0.37
40 grafted	1006	366	336	180	0.33

**Table 4:** Transversely isotropic elastic stiffness components of CNTs [GPa].

	SWNT					MWNT				
Grafting	*C*_11_	*C*_22_	*C*_12_	*C*_23_	*C*_55_	*C*_11_	*C*_22_	*C*_12_	*C*_23_	*C*_55_
0	699	365	158	154	300	1238	575	239	193	371
20	620	296	108	97	277	1145	557	194	187	350
40	567	282	86	89	241	1085	545	170	186	336

## Data Availability

Not applicable.
